# 

**Published:** 1893-08

**Authors:** 


### Communicated.

### WORLD'S COLUMBIAN DENTAL CONGRESS.

### Circular from the General Executive Committee.

*To the Dentists of the United States of America, Canada, Mexico, Central America, and South America, greeting:*

The movement to hold a Dental Congress in Chicago, Illinois, August 14-19, 1893, inclusive, received its official status from the joint action of the Southern Dental Association at its meeting in July, 1890, held at Atlanta, Georgia, and the meeting of the Missouri Dental Associ- held at Excelsior Springs, Missouri, in August, 1890. The undersigned General Executive Committee was appointed by the two associations to adopt rules and regulations, fix the time for convening the Congress, secure the place for holding the sessions, and make such other preliminary arrangements as it deemed necessary.

The work of appointing committees to promote the success of the Congress is finished, the permanent officers have been chosen, the honorary officers have been appointed in all foreign countries, and the time and place of meeting fixed.

A general invitation has been issued, asking the co-operation of the reputable dentists of the civilized world to meet with the dentists of the United States of America at the time and place fixed, for the presentation of papers, both scientific and practical, covering the entire range of theory and technology. It is believed that the newest investigations, discoveries, and methods in physiology, histology, bacteriology, pathology, oral surgery, chemistry, materia medica, therapeutics, orthodontia, operative dentistry, prosthesis, and deontology will be presented to this Congress in a manner not heretofore attempted in any international gathering of a similar character.

It is with pleasure, therefore, that we appeal to the dentists of America to assist in this great undertaking, which promises so much for the future of dentistry and dental surgery, in placing its practical and humanitarian objects before the public at large. This Congress will be an educator of such vast proportions to the practitioners of dentistry that few can realize the direct benefits which will accrue, not only to those participating, but to those who deny themselves the opportunity to make history for the generations yet to follow.

The Transactions, when printed, will be a permanent record of scientific development that may well serve as a starting-point in future professional advancement, education, legislation, and prophylaxis.

Nothing will be omitted to provide for the comfort and entertainment of those who lend their presence for the furtherance of the objects of this Congress, and a programme of such literary merit will be presented as shall reflect in the clearest manner the past history and present development of dental science, including also the practical demonstration of every phase of operations known. These demonstrations will be made by those best fitted by native ingenuity, education, and technical skill in bacteriology, histology, pathology, oral surgery, and other more directly practical subjects, such as orthodontia, prosthesis, electricity, and mechanical operations on the teeth, jaws and associate parts.

The facilities for meetings and clinical demonstrations are ample to accommodate all who are entitled to admission to the Congress. The Memorial Art Palace is situated near the center of transportation, it is isolated from traffic, and is well lighted and ventilated.

The general headquarters will be located at 300 Michigan Avenue, within ten minutes' walk of the assembly-rooms. All communications to the secretary of the General Executive Committee to be sent to this address after July 15th.

The profession in America must now assume the responsibility of making this Congress a success on the lines laid out by the General Executive Committee. This can only be accomplished by the immediate response of those who contemplate being present in person, or by contribution, financial or otherwise.

The committee urgently requests an immediate decision from those purposing to attend, in order to facilitate the work of the various departments and reduce to a reasonable certainty the attendance from America.

Contributions of money should be made directly and at once to the chairman of each State Finance Committee for transmission to the Treasurer, who will issue his receipt for the same. Accompanying this circular are the codified rules and regulations of the Congress and instructions for the guidance of all.

Read this circular carefully and preserve it for future reference. Adherents of the Congress will address letters of inquiry to the secretary of the General Executive Committee, in order to receive an official reply.

Cordially and fraternally yours,

W. W. WALKER, *Chairman of the General Executive Committee*, 67 W. Ninth St., New York City, N.Y.

A. O. HUNT, *Secretary of the General Executive Committee*, Iowa City, Iowa.

L. D. SHEPARD, *President of the Congress*, 330 Dartmouth St., Boston, Mass.

A. W. HARLAN, *Secretary-General of the Congress*, 1000 Masonic Temple, Chicago, Ill.

JOHN S. MARSHALL, *Treasurer*, Venetian Building, Chicago, Ill.

W. J. BARTON, Paris, Texas.

L. D. CARPENTER, Atlanta, Ga.

J. Y. CRAWFORD, Nashville, Tenn.

M. W. FOSTER, 9 Franklin St., Baltimore, Md.

H. J. MCKELLOPS, 2630 Washington Ave., St. Louis, Mo.

G. W. MCELHANEY, Columbus, Ga.

H. B. NOBLE, New York Ave., Washington, D. C.

JOHN C. STOREY, Dallas, Texas.

C. S. STOCKTON, Newark, N. J.

J TAFT, 122 W. Seventh St., Cincinnati, O.

*Members of the General Executive Committee.*

FINANCES.

Desiring that every reputable member of the dental profession shall be identified with the Congress,—

*Resolved*, That a payment of ten dollars ($10.00) shall entitle one to the Transactions and to membership, if eligible.

That a payment of twenty dollars ($20.00) shall entitle one to the Transactions and to membership as above, and to the commemorative medal.

That a payment of thirty dollars ($30.00) or upward shall have all the advantages of the twenty-dollar ($20.00) subscription, and also recognition as a contributor to the financial success of the Congress.

That any student presenting a certificate from the dean or secretary of a reputable dental college shall be entitled to student membership, and also to a copy of the Transactions, on the payment of five dollars ($5.00).

RULES AND REGULATIONS.

All public announcements for the General Executive Committee shall bear the signatures of both the chairman and the secretary.

The admission fee to the World's Columbian Dental Congress shall be fixed at ten dollars, to be collected only from residents of the United States.

All papers to be read before the Congress shall be in the hands of the Committee on Printing Transactions not later than July 1, and shall not exceed forty-five minutes in the time of presentation. Said committee shall have full power to accept or reject any paper, to revise or suggest a revision by the authors, and to publish or not in the Transactions the whole or parts of papers read, or abridgments thereof.

The official languages of the Congress shall be English, French, Spanish and German, and the papers shall be printed in the Transactions in the languages in which they are read.

After a paper has been accepted, the committee shall prepare a brief synopsis, to be published in the official languages of the Congress.

The chairman of each committee shall send reports of its progress to the chairman and secretary of the General Execurive Committee at such frequent intervals as will keep them informed of all the work accomplished.

All circulars issued by any committee must be sent to each member of the General Executive Committee, and they shall be of uniform size—viz., that of the minute forms issued by the secretary.

The Dental Congress offers a medal for the best popular paper on Dental Hygiene, for public distribution, to be referred to Committee No. 23, to be called the Committee on Prize Essays.

All matters of business presented at the general sessions of the Congress shall be referred to the General Executive Committee, and must receive the endorsement of the committee before they can be entertained by the President of the Congress.

The management of the World's Congress Auxiliary of the Columbian Exposition have offered suitable accommodations in the Memorial Art Palace, on the lake front, in Chicago, for the sessions of the World's Columbian Dental Congress, August 14, 1893.

### INVITATION.

The duties of the Committee on Invitation shall be to invite such scientific persons residing in the United States and foreign countries who are not members of the profession, but who by their recognized attainments in special departments of science would add interest to the meeting. They shall also have the authority to invite such dentists of high standing and reputation in foreign countries as may be agreed upon by a majority of the committee, and a card from the chairman of said committee to the chairman of the Committee on Registration shall be deemed evidence of the reputability of the holder thereof to entitle him to membership in the Congress, and they shall also furnish the Committee on Membership with a list of the names and residences of those invited.

### MEMBERSHIP.

The duties of the Committee on Membership shall be to pass upon all applications for membership which may be referred to it by the Committee on Registration or the Treasurer.

The membership shall consist of legally qualified and reputable dentists (as defined in the code of ethics of the American and Southern Dental Associations) residing in the United States, and such other scientific persons as may be invited by the Committee on Invitation, each and every member to be entitled to one copy of the Transactions.

All dentists residing in foreign countries who desire to acquire membership in the Congress will file their application with the honorary President or Vice-Presidents of their respective countries, who are empowered to pass upon their eligibility.

When applications are satisfactory to the honorary President or Vice-Presidents, or a majority of them, in said country, the names so agreed upon shall be transmitted by July 15, 1893, to the chairman of the Committee on Registration, who will proceed to issue a membership card without further reference.

### OFFICERS OF THE CONGRESS.

### *Science—Department A.*

SECTION 1.—*Anatomy and Histology.*—Chairman, R. R. Andrews, Cambridge, Mass.; vice-chairman, E. P. Beadles, Danville, Va.; secretary, F. T. Breene, Iowa City, Iowa.

SECTION 2.—*Etiology, Pathology and Bacteriology.*—Chairman, G. V. Black, Jacksonville, Ill.; vice-chairman, Geo. S. Allen, New York, N. Y.; secretary, E. S. Chisholm, Tuscaloosa, Ala.

SECTION 3.—*Chemistry and Metallurgy.*—Chairman, D. R. Stubblefield, Nashville, Tenn.; vice-chairman, J. S. Cassidy, Covington, Ky.; secretary, E. V. McLeod; New Bedford, Mass.

SECTION 4.—*Therapeutics and Materia Medica.*—Chairman, F. J. S. Gorgas, Baltimore, Md.; vice-chairman, N. S. Hoff, Ann Arbor, Mich.; secretary, Geo. E. Hunt, Indianapolis, Ind.

*Applied Science—Department B.*

SECTION 5.—*Dental and Oral Surgery.*—Chairman, T. W. Brophy, Chicago, Ill.; vice-chairman, M. H. Cryer, Philadelphia, Pa., secretary, J. F. Griffiths, Salisbury, N. C.

SECTION 6.—*Operative Dentistry.*—Chairman, Wm, Jarvie, Brooklyn, N. Y.; vice-chairman, Daniel N. McQuillen, Philadelphia, Pa.; secretary, Henry W. Morgan, Nashville, Tenn.

SECTION 7.—*Prosthesis, Orthodontia.*—Chairman, C. L. Goddard, San Francisco, Cal.; vice-chairman, T. S. Hacker, Indianapolis, Ind; secretary, E. H. Angle, Minneapolis, Minn.

SECTION 8.—*Education, Legislation, Literature.*—Chairman, J. J. R. Patrick, Belleville, Ill.; vice-chairman, H. L. McKellops, San Francisco, Cal.; secretary, W. H. Whistler, Cleveland, Ohio

---

### RESOLUTIONS ADOPTED BY THE CHICAGO DENTAL SOCIETY ON THE DEATH OF DR. W. W. ALLPORT.

WHEREAS, In the death of Dr. W. W. Allport, a leader in our profession has fallen, and as a mark of our appreciation of his services and skill, be it

*Resolved*, That in his death the dental profession has lost a member whose extraordinary skill as an operator placed him among the foremost dentists of the world. His work in promoting the highest interests of the profession will ever be conspicuous, and the prosperity enjoyed by younger members is due in a great measure to his achievements.

*Resolved*, That a copy of this preamble and resolution be sent in proper form to the family of the deceased, and also to the dental journals for publication.

TRUMAN W. BROPHY,<br>

A. W. HARLAN,<br>

J. N. CROUSE, } Committee.

### TWENTY-FOURTH ANNUAL MEETING OF THE CALIFORNIA STATE DENTAL SOCIETY.

The twenty-fourth annual meeting of the California State Dental Association was held at the College of Dentistry, San Francisco, on June 13 to 16 inclusive.

The following officers were elected for the ensuing year: President, Luther A. Teague, 10 Geary street, San Francisco; 1st vice-president, J. W. Hays, Jr., Grass Valley; 2nd vice-president, C. S. Goddard, San Francisco; 3rd vice-president, W. F. Lewis, Oakland; recording secretary, W. J. King, San Francisco; corresponding secretary, C. E. Post, 14 Grant Ave., San Francisco; Treasurer, T. N. Iglehart, San Francisco.

CHAS. E. POST, D. D. S.,
Cor. Sec'y.

---

### ANNUAL MEETING OF THE NATIONAL ASSOCIATION OF DENTAL FACULTIES.

The meeting will be held in the house of the Columbian Dental Club, Chicago, No. 300 Michigan Avenue, beginning on Thursday, August 10th, at 10 o'clock a. m., and continue through that and the succeeding day. It is important that all matters of business to come before that meeting be properly prepared beforehand, so that business can go promptly forward. It is to be hoped that all persons interested will give special attention to this request, and that every member be promptly present at the beginning of the meeting, as only the two days will be available for the work.

J. TAFT,

FRANK ABBOTT,

A. O. HUNT

} Executive Com.

### To Readers and Correspondents.

Communications intended for publication, and exchange journals and papers, should be addressed to the Editor of The American Journal of Dental Science, No. 845 N. Eutaw St. Baltimore, Md. All letters relating to the business of the Journal, or containing cash remittances, to be directed to Messrs. Snowden & Cowman, No. 9 W. Fayette Street, Baltimore, Md. Articles for publication should be received by the Editor at least two weeks previous to the first of the month on which the number in which it is designed for them to appear, is issued.

☞ The American Journal of Dental Science will contain fifty pages of reading matter in each number, making a volume of about Six Hundred pages,

EXCLUSIVE OF ADVERTISEMENTS

